# Outlines of Modern Chemistry, Etc.

**Published:** 1878-01

**Authors:** 


Outlines of Modern Chemistry, Organic, Based in Part upon Rich's Manuel de Chimie. By Gilbert Wheeler, Prof. of Chemistry in the University of Chicago, and formerly Prof. of Organic Chemistry in the Chicago Medical College. Jansen, McClure & Co. 1877.

This volume of 220 pages treats of organic chemistry exclusively, as the title indicates. It is not, therefore, a thorough work on the general science of chemistry, but more practical and useful for the study of the department of chemistry of which it treats, than any general treatise on chemistry with which we have met. To such students of chemistry, then, or medical men, as wish to peruse a work exclusively devoted to the action and reaction of purely organic substances, we recommend this book. Vegetable or organic acids, alcohols, ethers, alkaloids, etc., are particularly alluded to, and in an interesting manner.

